# Comparative Evaluation of the Activity of Various Lentiviral Vectors Containing Three Anti-HIV Genes

**DOI:** 10.3390/microorganisms11041053

**Published:** 2023-04-18

**Authors:** Olga Vladimirovna Orlova, Dina Viktorovna Glazkova, Ramil Rafailovich Mintaev, Galina Mikhailovna Tsyganova, Felix Anatolevich Urusov, German Alexandrovich Shipulin, Elena Vladimirovna Bogoslovskaya

**Affiliations:** Federal State Budgetary Institution “Centre for Strategic Planning and Management of Biomedical Health Risks” of the Federal Medical Biological Agency, 119121 Moscow, Russia

**Keywords:** lentiviral vectors, TRIM5a, human immunodeficiency virus type 1 (HIV-1), C-peptide, miRNA

## Abstract

A promising direction in the treatment of HIV infection is a gene therapy approach based on the insertion of antiviral genes aimed at inhibiting HIV replication into the genome of host cells. We obtained six constructs of lentiviral vectors with different arrangements of three antiviral genes: microRNAs against the CCR5 gene, the gene encoding the C-peptide, and the gene encoding the modified human TRIM5a protein. We found that despite containing the same genes, these vectors were produced at different titers and had different effects on cell viability, transduction efficiency, and expression stability. Comparative evaluation of the antiviral activity of three of the six developed vectors that showed stable expression was carried out using the continuous SupT1 lymphocytic cell line. All of the vectors protected cells from HIV infection: the viral load was several orders of magnitude lower than in control cells, and with one vector, complete cessation of virus growth in modified cells was achieved.

## 1. Introduction

One of the promising strategies in the development of drugs for the treatment of HIV infection is a gene therapy approach based on the insertion of genes that protect against viral infection into the genome of host cells [1,2]. The target cells are usually CD4+ T lymphocytes, the main population of cells affected by HIV, or their precursors, i.e., hematopoietic stem cells (HSCs). Currently, lentiviral vectors (LVs) are considered the most effective means of gene delivery, ensuring the integration of genes into the cellular genome. Integration is important when working with proliferating immune cells because it guarantees the transfer of genes to daughter cells and their protection from the virus [3,4,5].

To date, many genes with anti-HIV activity have already been proposed, some of which have shown high efficiency in preclinical studies and have even been evaluated in clinical studies [6]. Of particular interest are genes with products that act at the early stages of the HIV life cycle and prevent the integration of the virus into the cell genome; such genes are considered potentially highly effective [7]. Among them, genes aimed at turning off the CCR5 chemokine receptor (which is necessary for penetration of the R5-tropic virus), the C-peptide (which inhibits the fusion of HIV with a cell), and the modified human TRIM5a protein are notable.

Three cases of curing HIV infection associated with bone marrow transplantation from donors carrying a non-functional CCR5∆32 gene [8] evidence that transplantation of immune cells with the disabled CCR5 gene is safe and can be used for HIV treatment. Many options have been proposed to knockdown the CCR5 receptor gene in patient cells [8], including the use of RNA interference. For example, we previously described a tandem miRNA that can effectively enhance expression of the CCR5 gene [9] and inhibit HIV replication in T lymphocytes containing such a tandem [10].

C-peptides are 36–46 aa peptides with a structure similar to that of the C-terminal helical domain of the HIV-1 gp41 protein. C-peptide interacts with the HIV Env protein, disrupting its structure and preventing the virus from merging with a cell. One of the promising C-peptides is the V2O peptide with reduced immunogenicity and high activity against a wide range of HIV strains [11]. A membrane-anchored form of C-peptide ensures its expression on the cell surface and is shown to be the most effective for the gene therapy of HIV infection [7,11].

The TRIM5a protein is an intracellular factor involved in protection against retroviruses. The spectrum of retroviruses neutralized by TRIM5a is specific for different mammalian species. Rhesus monkey TRIM5a completely protects cells from HIV infection, in contrast to human TRIM5a [12]. It has been shown that the TRIM5a-HRH chimeric protein, in which 11 amino acids of human TRIM5a are replaced by 13 amino acids from TRIM5a of rhesus monkey, is able to effectively inhibit HIV replication [12,13,14]. Importantly, this protein acts before the retrovirus enters the nucleus and prevents the insertion of proviral DNA into the genome.

It is advisable to use several antiviral genes simultaneously, as suppression of the virus at different stages of its life cycle increases effectiveness and prevents formation of gene-resistant variants of the virus. In the present work, six variants of a lentiviral vector that carried three genes, including a tandem of artificial microRNAs against the CCR5 gene, the TRIM5a-HRH gene, and the gene encoding the C-peptide, were generated and characterized, and their antiviral activity was studied in vitro.

## 2. Materials & Methods

### 2.1. Cell Lines Used

Human embryonic kidney cells HEK293FT (ThermoFisher, Waltham, MA, USA) and human SupT1 T lymphoblasts (ATCC CRL-1942) were used in this work.

HEK293FT human embryonic kidney cells were cultured in standard DMEM containing 10% fetal bovine serum (FBS) (HyClone, Logan, UT, USA); 4 mM L-glutamine (Gibco, Franklin Lakes, NJ, USA); 1 mM sodium pyruvate (Gibco, USA); 0.1 mM essential amino acids MEM NEAA 100× (Gibco, USA); and streptomycin and penicillin at concentrations of 100 μg/mL and 100 units/mL, respectively (Gibco, USA). Suspension cells of the SupT1 lymphoblastoid line were cultured in Advanced RPMI medium (Gibco, USA) with the addition of 2% FBS. All cultures were incubated at a constant temperature of 37 °C and a CO_2_ content of 5% in air.

### 2.2. Plasmid Constructs

Sequences encoding the TRIM5a-HRH gene, miRNAs, pPGK and Ef1a promoters, and the EGFP reporter gene were amplified, using previously obtained plasmids as templates. For the TRIM5a-HRH, GFP, pPGK, and Ef1a genes, we used the pT plasmid [14]; for the mic13lg + mic1002 gene, we used the plasmid mic13lg + mic1002-Puro [10]. To generate a sequence of the V2O gene coding membrane-anchored peptide, a transmembrane domain and a secretion signal sequence were added to the codon-optimized sequence of the V2O-peptide described in the article by L. Egerer et al. [11]. For codon optimization, the OPTIMIZER web service (http://genomes.urv.es/OPTIMIZER, accessed on 1 March 2021) and a codon frequency table were used (https://www.kazusa.or.jp/codon/cgi-bin/showcodon.cgi?species=9606, accessed on 1 March 2021). The V2O gene was assembled by overlap extension PCR, resulting in the pV2O plasmid.

The primers and fragments used to generate LV N1–LV N6 plasmids are shown in Table S1 (Supplementary).

All genetic engineering manipulations were performed according to standard protocols [15]. Isolation of plasmid DNA for transfection of HEK293FT cells was performed using the Plasmid Maxi Kit (QIAGEN) under sterile conditions.

### 2.3. LV Particles Production

The second-generation packaging system [14] consisting of the two packaging plasmids pCMV-dR8.91 (encoding the HIV proteins GAG, POL, TAT, and REV) and pCMV-VSV-G (encoding the envelope glycoprotein of the vesicular stomatitis virus VSV-G) was used to produce LV particles [14].

The day before transfection, HEK293FT cells were seeded at a density of 0.7 × 10^5^ cells/cm^2^ into 2 Hyperflask (Corning, New York, NY, USA). The ratio between the plasmid DNA of the vector and the DNA of the packaging plasmids pCMV-dR8.91 and pCMV-VSV-G was 6:6:1. A solution of linear polyethyleneimine PEI MAX 40000 (Polysciences, Niles, IL, USA) was used for transfection. DNA samples (238.3 μg) in 17,200 μL of 150 mM NaCl were mixed with PEI working solution, which was prepared by diluting 953 μL of PEI stock solution (1 g/L) to 17,200 μL with 150 mM NaCl solution. The resulting mixture was transfected into each Hyperflask. The day after transfection, the medium was replaced with serum-free OptiMEM (Gibco, USA). The virus suspension was collected at 72 h after transfection and centrifuged at 3500 rpm for 5 min using a CM-6MT centrifuge (Elmi, Riga, Latvia). The supernatant was filtered through a 0.45 µm PES filter (Corning, USA) and incubated with 25 U/mL benzonase (Merc, Hackensack, NJ, USA) at 37 °C for 1 h.

### 2.4. Tangential Flow Filtration and Ultracentrifugation

The concentration in tangential flow was performed with a KrosFlo KR2i device (Repligen, Waltham, MA, USA) and a column with a PES filter and pore diameter of 500 kDa preliminarily equilibrated with phosphate-buffered saline (PBS) and 1% fetal bovine serum (FBS). Then, it was dialyzed against phosphate-buffered saline (PBS) with 1% FBS.

The concentrated viral suspension was ultracentrifuged (UC) through a cushion of 2 mL of 20% sucrose solution for 2 h at a speed of 25,000 rpm at a temperature of +4 °C using an Optima XPN-100 ultracentrifuge (“Beckman Coulter”, Brea, CA, USA). After UC, the virus pellets were resuspended in 600 µL of RPMI growth medium.

### 2.5. Determination of Infectious Titer

The infectious titer of the LV particles (transduction units/mL, or TU/mL) was determined using the SupT1 cell line. The cells were transduced with a series of dilutions of freshly thawed lentiviral particles in growth medium supplemented with 2 μg/mL of polybrene. The next day, the growth medium was replaced with fresh medium. The titer was determined 48 h after transduction by the percentage of the fluorescent EGFP+ cells in the population, using a Novo-Cyte Quanteon flow cytometer (Agilent Technologies, Santa Clara, CA, USA).

### 2.6. Transduction of Cells by LV Particles

SupT1 cells were plated in a 24-well plate at a concentration of 2 × 10^5^ cells/mL in 0.5 mL per well. Then, viral particles were added in the amount necessary to obtain the desired multiplicity of infection (MOI). The cells were incubated overnight in the presence of polybrene (2 μg/mL) at 37 °C in a CO_2_ incubator. The next day, the growth medium was replaced with fresh medium.

### 2.7. Determination of Cell Viability

The number of viable and dead cells in suspensions was determined by staining with trypan blue (Sigma, St. Louis, MO, USA). An equal volume of the cell suspension was added to 25 µL of PBS with 0.4% trypan blue and mixed. After 2 min, the number of blue cells (dead cells) and transparent cells was counted using a Luna II cell counter (Logos Biosystems, Anyang, Republic of Korea). The percentage of live and dead cells was also calculated by a cell counter.

### 2.8. Measurement of the Percentage of Transduced Cells by Flow Cytometry

Before flow cytometry, cells were stained with a LIVE/DEAD™ Fixable Violet Dead Cell Stain Kit (Invitrogen, Waltham, MA, USA), precipitated, and diluted in PBS to a density of 1 × 10^6^ cells/mL at a rate of 1 µL of staining solution per 1 mL of cell suspension. Staining was carried out for 30 min at +4 °C. The percentage of EGFP+ fluorescent cells in the living cell population and the mean fluorescence intensity (FI) were determined using a NovoCyte Quanteon flow cytometer (Agilent Technologies, USA) in the FITC channel. The measurement results were processed using NovoExpress 1.4.1 software (Agilent Technologies, USA).

### 2.9. Measurement of Vector Copy Number and Quantification of V2O and TRIM5a-HRH Mrna Abundance in Supt1 Cells

SupT1 cells transduced with various lentiviral vectors were cultured for 3 weeks to remove residual plasmid DNA introduced into the culture along with the lentiviral particles. Then, 10^5^ cells of each sample were collected and treated with Benzonase (Merck, Darmstadt, Germany). Nucleic acid extraction was carried out by precipitation with isopropanol, using the AmpliTest RIBO-prep reagent kit (Centre for Strategic Planning of FMBA, Moscow, Russia) according to the instructions. The integrated vector copy was determined by TaqMan quantitative PCR (qPCR) with the primers ccttgtataaatcctggttgctgtct and ggaaaggagctgacaggtggt and the probe R6G-tcaggcaacgtggcgtggtgtg-BHQ2 that target the wpre element. The human β-globin gene copy number was detected using the primers gtcagggcagagcctctattgct and ccacatgcccagtttctattggtct and the probe Fam-tgcccaggggcctcaccacca-BHQ1. The vector copy number was normalized to the human β-globin gene copy number, obtaining the average number of vector copies per cell.

To quantify the V2O and TRIM5a-HRH mRNA abundance, one-step reverse transcription qPCR (RT-qPCR) was performed using in-house reagents and the following primers and probes: for TRIM—primers gacaagtgagctctccgaaacca and gagagcccaggatgccagtaca, probe R6G—accctgttcaccttccccagcctgac-BHQ1; for V2O—primers gctgattgcgctggtgacca and cggttccagttccaggcgt, probe R6G—tgctggcggtgctgggcattaccg-BHQ1. The primers specified for TRIM detected only the transgenic TRIM5a-HRH gene. The human β-glucuronidase (GUS) gene was used as a control gene for the normalization of gene-expression levels (primers catttggaattttgccgatttcat and gtttcattggcaatcttccagtatct and probe Fam-cggcagagacaaccaaaaagtgcag-BHQ1). Before RT-qPCR, the samples were treated with DNase (DNase-1, Invitrogen, USA).

### 2.10. Determination of the HIV p24 Antigen

The amount of p24 antigen in samples was measured using the HIV-1 p24-antigen-ELISA-BEST kit (Vector BEST, Novosibirsk, Russia). To quantify the concentration of p24 antigen in the samples, standard samples (calibrators) with known p24 antigen concentrations were analyzed in each run. Calibrator samples were obtained by diluting recombinant HIV-1-p24 capsid protein (HIV-1-p24 Antigen AG 6054, Aalto Bio Reagent, Ireland).

### 2.11. HIV-1 Production and Cell Infection

Plasmid pNL4-3, containing the full-length genome of the NL4-3 HIV strain, was obtained from the United States under the NIH AIDS Research and Reference Reagent Program. To obtain the virus, HEK 293FT cells were transfected with the corresponding plasmid. After 2 days, the culture liquid containing infectious viral particles was collected, and the concentration of the p24 antigen was determined.

First, 25 µL (0.35 ng) of the NL4-3 virus was added to 1 mL of SupT1 cell suspension in Advanced RPMI + 2% FBS medium at a density of 10^6^ cells/mL. Then, the cells were incubated at 37 °C for 2 h, with stirring every 30 min; after 2 h, the cells were washed twice with fresh Advanced RPMI medium without serum, and Advanced RPMI + 2% FBS was added to a density of 10^6^ cells/mL. On the 2nd, 4th, 6th, 10th, 13th, 17th, 20th, and 23rd days, the cells were counted and subcultured to a density of 0.5 × 10^6^ cells/mL. On the same days, samples of culture media were taken for p24 antigen concentration analysis.

### 2.12. Statistical Data Processing

Mean values and the standard deviation (SD) were calculated using Microsoft Excel 2016 software (Microsoft Corporation, Redmond, DC, USA).

## 3. Results

### 3.1. Design of Lentiviral Vectors Containing Three Anti-HIV Genes

**Figure 1:**
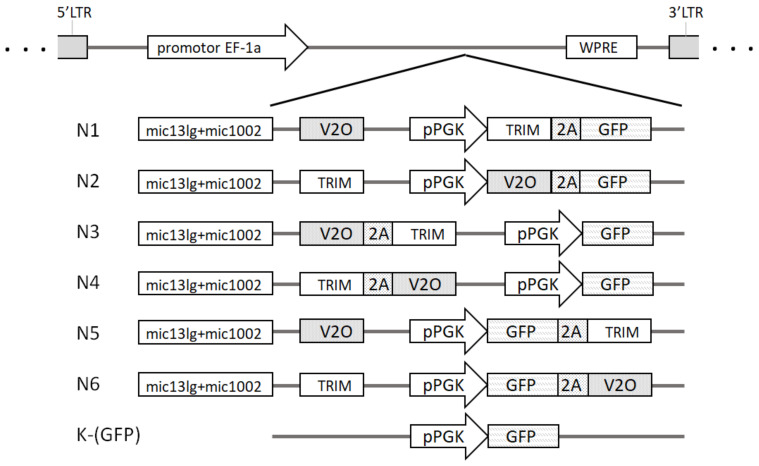
Scheme of the lentiviral vectors. Designations: 5′LTR and 3′LTR—5′ and 3′ long terminal repeats of HIV (long terminal repeats); WPRE—woodchuck hepatitis virus posttranscriptional regulatory element, EF-1a—elongation factor-1 alpha gene promoter, pPGK—phosphoglycerate kinase gene promoter, 2A—protein chain self-separation peptide.

The following anti-HIV genes were used: the mic131g-mic1002 gene encoding a tandem of two artificial microRNAs against CCR5; the TRIM5α-HRH gene encoding the modified human TRIM5α protein; and the V2O gene encoding a C-peptide, a fusion inhibitor. As gene expression can be affected by the arrangement of genes in a vector, as well as the elements regulating their expression, six different LVs were produced, each containing genes and regulatory elements in a different order (Figure 1). The genes were divided into two reading frames, each of which was under the control of its own constitutive promoter. The first frame was under the control of the elongation factor-1 alpha (EF1α) gene promoter; the second was under the control of the phosphoglycerate kinase (pPGK) gene promoter. One of the promoters regulated two proteins separated by a short 2a peptide (T2A derived from *Thosea asigna* virus), and this configuration allowed for a reduction in the size of the vector. In addition to antiviral genes, each construct included a marker gene encoding the green fluorescent protein GFP. In all constructs, the mic13lg + mic1002 miRNA tandem was located immediately after the EF1α promoter, before the open reading frame encoding the V2O or TRIM5α-HRH gene (hereinafter referred to as TRIM).

### 3.2. Efficiency of Lentiviral Vector Production

For all described vectors, plasmid constructs were obtained and used to produce LV particles, together with the control vector K-(GFP). The titer of the obtained particles was evaluated before and after their concentration and purification.

**Figure 2:**
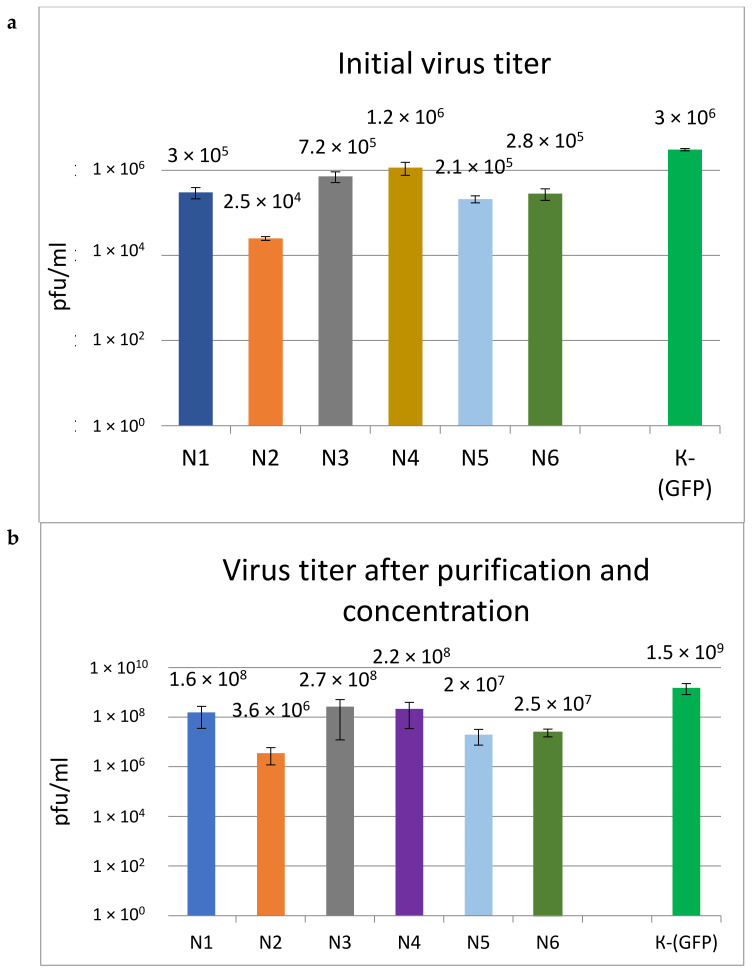
Comparison of titers of different LVs. (**a**)—Initial titer of viruses. (**b**)—Virus titer after purification and concentration. Columns reflect the average value of the +−SD (*n* = 4) titer of LV particles.

According to the results presented in Figure 2a, the LV titers differed significantly, ranging from 2.5 × 10^4^ to 1.2 × 10^6^ infectious particles per ml, and were 2.5–120 times lower than the titer of the control GFP vector. The titers of the N3 and N4 vectors were the highest; for the N1, N5, and N6 vectors, average titers were obtained. The lowest titer of 2.5 × 10^4^ was obtained for N2. After purification and concentration, LV titers increased by approximately two orders of magnitude (Figure 2b), and their ratio remained similar.

### 3.3. Transduction Efficiency, Transgene Stability in Cell Culture, and Influence of Different LV on the Viability of SupT1 Cells

**Figure 3:**
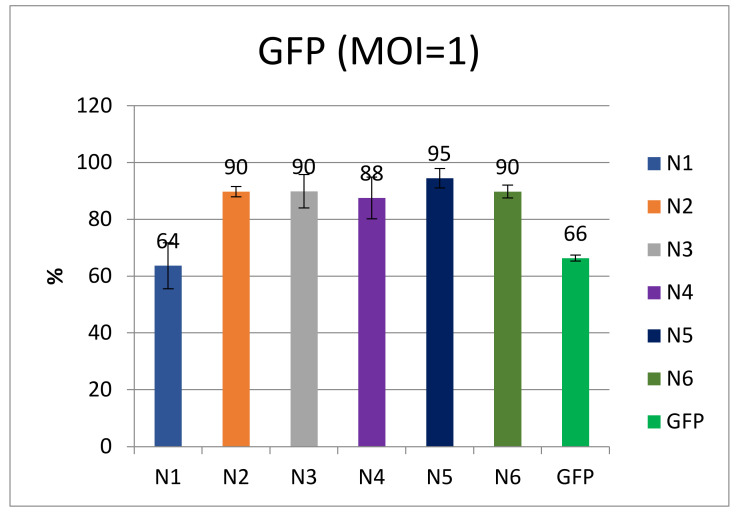
Comparison of the efficiency of transduction by different LVs at MOI = 1 (*n* = 3).

The transduction efficiency, transgene stability, and viability of transduced cells were evaluated using SupT1 cells, which were transduced with the test vectors at an MOI of 1 and cultured for 5 weeks. The transduction efficiency was assessed at 4 days after transduction (Figure 3).

As shown in Figure 3, the transduction efficiency of most vectors was 88–95%. The lowest efficiency was observed for N1, but was comparable to the control.

**Figure 4:**
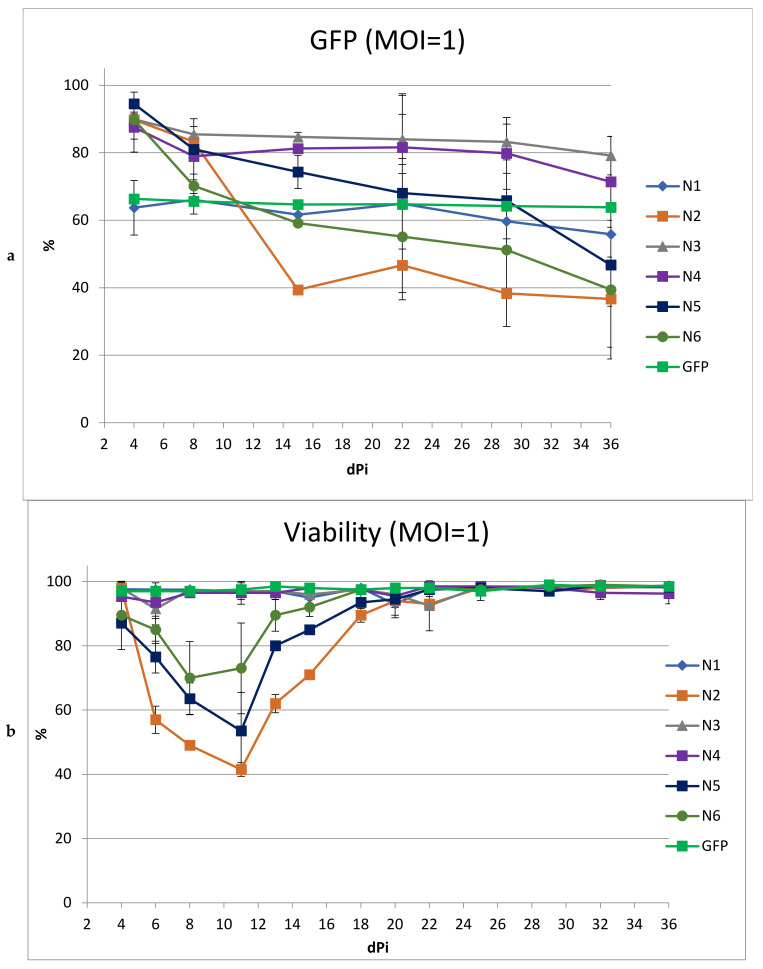
Analysis of SupT1 cells after transduction with different LVs. SupT1 cells were infected with LVs at MOI = 1. Then 2–3 times a week, the percentage of GFP-positive cells and the cell viability were measured. (**a**) Percentage of GFP-positive cells in culture within 5 weeks after transduction and (**b**) Cell viability within 5 weeks after transduction; *n* = 3.

The stability of the transgenes in culture was assessed by the dynamics of the changes in the proportion of cells containing the vector in culture over time (Figure 4a). For some of the LVs, a pronounced drop in the percentage of GFP+ cells was observed. For the N2 vector, the percentage of transduced cells decreased by 50% in 15 days; for N5 and N6, the decrease in the proportion of GFP+ cells was slower, but also reached 47 and 50%, respectively, by day 36. The decrease in the percentage of GFP+ cells was accompanied by low cell viability in culture (Figure 4b). This likely indicates the cytotoxicity of these vectors, which leads to the death of cells containing the LVs. For cells transduced with N1, N3, and N4, the drop in % of GFP+ cells was weakly pronounced and amounted to 8, 10, and 15%, respectively, with the main decrease occurring in the first week. This initial slight decrease may be due to the fact that the vector failed to integrate into some of the cells and was lost rapidly during cell division. No negative impact on viability was observed.

**Figure 5:**
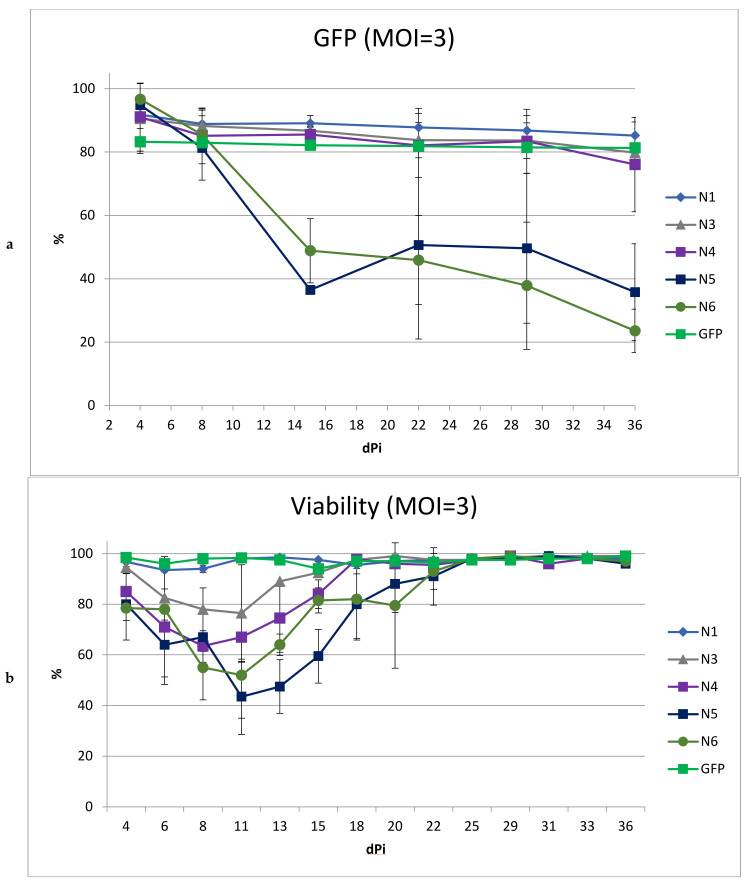
Analysis of SupT1 cells after transduction with different LVs. SupT1 cells were infected with LVs at MOI = 3. Then 2–3 times a week, the percentage of GFP-positive cells and the cell viability were measured. (**a**) Percentage of GFP-positive cells in culture within 5 weeks after transduction and (**b**) Cell viability within 5 weeks after transduction; *n* = 3.

To obtain a higher percentage of cells carrying the vector, including after long-term cultivation, we performed transduction with the same LVs using MOI = 3 (Figure 5). We did not include the N2 vector in this experiment due to its low titer and high toxicity. At MOI = 3, the transduction efficiencies for all LVs were comparable and ranged from 83 to 96%.

As expected, an increase in MOI led to a greater drop in viability in cultured cells transduced with N5 and N6. Moreover, the decrease in the percentage of cells with the vector during cultivation was even more pronounced than with MOI = 1. In cultures transduced with vectors N1, N3, and N4, the dynamics of the decrease in the percentage of GFP+ cells repeated the dynamics of the decrease when using MOI = 1, decreasing by the end of the experiment by 7%, 11%, and 15%, respectively. Additionally, viability analysis revealed a negative effect on the cells by the N3 and N4 vectors, but not the N1 vector; thus, the use of MOI = 3 for transduction revealed the advantage of N1 as a vector with reduced toxicity.

Three weeks after transduction (MOI = 1), we measured the levels of V2O and TRIM5a-HRH mRNA, as well as the average vector copy numbers in the cell cultures. The amount of mRNA was normalized to the average vector copy number. The TRIM5a-HRH expression did not differ for cells transduced with different vectors. For V2O, a significant difference was found only between the N1 and N3 transduced cell lines (Figure S1). For N1, the expression level was lower than for N3.

### 3.4. Comparisons of Antiviral Activity of LVs in SupT1 Cell Culture

**Figure 6:**
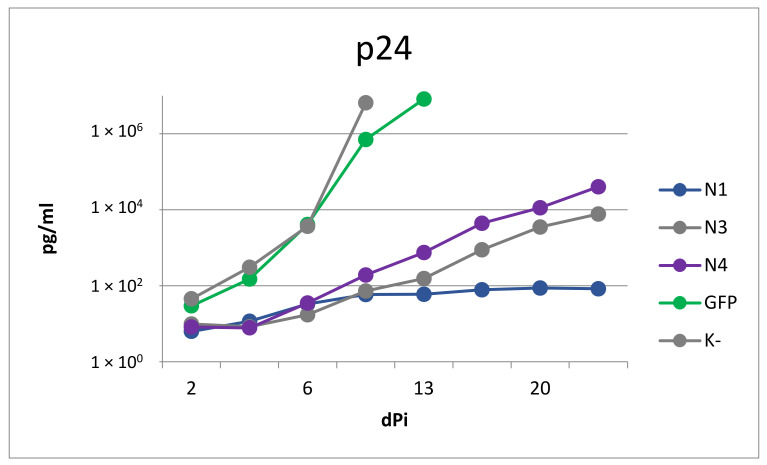
Dynamics of changes in the concentration of HIV p24 antigen in SupT1 cells transduced with N1, N3, N4, and GFP vectors and in the control non-transduced culture (K−).

LVs N1, N3, and N4, which showed the lowest toxicity, were selected for evaluation of the antiviral activity. At 10 days after transduction with N1, N3, and N4 vectors at a dose of 1 MOI, SupT1 cells were infected with the HIV-1 NL4-3 strain. The cells were observed for three weeks after infection to determine the growth rate of the virus via the p24 antigen accumulation in the growth medium (Figure 6).

The percentages of transduced cells for N1, N3, N4, and GFP were 71, 74, 78, and 95%, respectively, at 5 days before infection. In the cultures of non-transduced cells and cells transduced with the control GFP vector, an increase in the concentration of p24 antigen was observed, which indicated the active growth of the virus, leading to cell death on the 8th and 11th days after infection, respectively. In cells transduced with the vectors N1, N3, and N4, the virus growth rate was significantly reduced compared to control cells. Overall, the N1 vector provided maximum protection against the virus: after the 6th day, the amount of p24 antigen in the cell culture did not increase appreciably. The slight increase in the amount of p24 antigen on the 6th day after infection can be explained by virus replication in non-transduced cells; after the elimination of this factor, a tendency toward a reduced concentration of the antigen in the medium, caused by its slow degradation, occurred.

## 4. Discussion

The currently used antiretroviral therapy for HIV infection restrains development of the disease, but does not lead to a cure; hence, the search for new approaches to HIV therapy, one of which is gene therapy, is relevant. More than a dozen different antiviral genes have been proposed, the effectiveness of which has been demonstrated in vitro. However, one challenge of fighting HIV is its high variability, leading to the formation of variants that are resistant to individual genes [16]. As with the use of antiretroviral chemicals, this problem can be solved by combining several anti-HIV genes in a gene therapy drug. In addition, inclusion of genes that suppress virus replication at different stages of the life cycle in one vector increases antiviral activity. Therefore, we attempted to create a lentiviral vector for gene therapy that includes several genes. Three genes (anti-CCR5 microRNA gene; TRIM5a-HRH gene encoding a modified human TRIM5a protein; and V2O gene encoding C-peptide, a fusion inhibitor) that inhibit the virus at the initial stages of its life cycle and prevent insertion of proviral DNA were selected, as such genes can more effectively protect against infection [10,11,12].

Lentiviral vectors are currently the most efficient way to deliver genes to target cells. However, obtaining a vector that includes several genes can present certain problems [17]. It should be noted that to obtain a high percentage of transduced primary cells (lymphocytes and HSCs), it is necessary to use a high multiplicity of infection. As this is possible only at high titers of the vector, the production efficiency is an important parameter that further determines the feasibility of the practical use of LVs.

We constructed six lentiviral vectors containing the same three anti-HIV genes located in a different order and differentially regulated using promoters and the 2a peptide sequence. Analysis of the properties of these vectors showed that during production under the same conditions, the titers differed by more than 40 times, ranging from 2.5 × 10^4^ to 1.2 × 10^6^, and were 2.5–120 times lower than that of the control GFP vector.

The reduction in vector titer compared to the control vector was predictable and could be due to several factors. These include an increase in LV size [18]; the influence of the TRIM5a protein, which can lead to rapid degradation of Gag capsid proteins, suppressing virion assembly [19,20]; and promoter interference [13,21]. It has recently been shown that the Ef1a promoter in a similar vector adversely affected the titer, presumably by suppressing the promoter in the LTR, which regulates vector RNA production. Interference between Ef1a and the downstream PGK promoter is also possible [20]. For example, in constructs N3 and N4, the distance between Ef1a and pPGK is greater than in the other constructs, and the vectors obtained using these constructs showed a maximum titer.

In addition to the difference in the titer, the vectors differed in their effect on cell viability, which in turn led to a decrease in the proportion of cells containing the vector during cultivation. The toxicity of some vectors can be caused by various reasons. Antiviral proteins can affect cell viability, for example, high expression of the TRIM5a gene was shown to affect cell viability [22]. Therefore, the variation in viability among the cells transduced with different vectors could potentially be related to variations in gene expression. However, we were unable to see differences in the mRNA levels of the Trim5a-HRH and V2O genes in SupT1 cells transduced with different vectors. Unfortunately, we could not determine the amount of transgenic TRIM5a-HRH protein by a Western blot assay due to a low HRH-TRIM5a expression level in SupT1 cells, which cannot be distinguished from the background expression of the endogenous human Trim5a [20]. It can be assumed that some hybrid protein products resulting from incomplete cleavage of the 2a peptide may be toxic. It cannot be ruled out that the toxicity might be caused by a low titer of the vector, as it was necessary to add more vector, the preparation of which could contain impurities that negatively affect viability. It is notable that the vectors with the lowest titer after concentration and purification had the highest toxicity. In general, it was shown that at MOI = 1, vectors N1, N3, and N4 did not affect cell viability; at MOI = 3, only the N1 vector was nontoxic.

Analysis of the N1, N3, and N4 vectors’ antiviral activity showed the N1 vector to be the most promising for further research, as it provided maximum cell protection with minimal toxicity. To understand the potential value of the resulting lentiviral vector for HIV therapy, further studies of the vector in vitro on primary human lymphocytes and in vivo in a humanized mouse model are needed.

## Data Availability

The data will be available upon reasonable request to the corresponding authors.

## Supplementary Materials

The following supporting information can be downloaded at: https://www.mdpi.com/article/10.3390/microorganisms11041053/s1, Figure S1: Estimation of the mRNA level of the V2O and TRIM5a-HRH genes. The cells were transduced with various lentivectors, after 3 weeks the cells were collected, treated with benzonase, then mRNA was isolated and OT-qPCR was performed with primers to the V2O(A) and TRIM(B) genes. Normalization was performed on the average number of vector per cell, which was determined using qPCR as WPRE normalized to Globin. * *p* ≤ 0.05; Table S1: Plasmids and primers used to obtain LVs.
