# Supplementary material for: Comparative Evaluation of the Activity of Various Lentiviral Vectors Containing Three Anti-HIV Genes
Source: Microorganisms. 2023 Apr 18;11(4):1053. doi: 10.3390/microorganisms11041053 (PMC10141122; doi:10.3390/microorganisms11041053)
Supplement: Supplementary file 1 [file microorganisms-11-01053-s001.zip › microorganisms-2317393-supplementary.pdf]

Table S1 - Plasmids and primers used to obtain LVs.

LV	Plasmids and restriction enzymes used to obtain a plasmid vector	Plasmids used as a template for amplification of vector fragments (vector element)	Primer sequence 3'-5' used to obtain fragments
N1	mic13lg+mic1002-Puro (NheI-NotI)	pV2O (V2O gene)	V2O-NheI-F: actgctagctgtagaactccagcttagatcccgcgactctaggatcc gccgccaccatgggcgcgggc<br>V2O-XbaI-R: tatctagattacggttccagttccaggcg
		pT (pPGK)	pPGK-XbaI-F: taatctagataattctaccgggtaggggaggc<br>pPGK-SacII-R: gtggccgcgggaaaatagatctcgaaaggcccggagatgag
		pT (TRIM5a-HRH)	trim-SacII-F: tcccgcggccaccatggcttctggaatcctggttaatg<br>trim-XhoI-R: ctcctcgagagagcttggtgagcacagagtc
		pT (EGFP gene)	2a-EGFP-XhoI-F: tctctcgaggagggcagaggaagtcttctaacatgcggtgacgtgg aggagaatcccggccctggtaccgtgagcaagggcgaggag<br>EGFP-NotI-R: gacgcggccgccttgtacagctcgtccatgccga
N2	mic13lg+mic1002-Puro (NheI -NotI)	pT (TRIM5a-HRH)	trim-NheI-F:gccgctagcgccaccatggcttctggaatcctg<br>trim-Xho-R: ctcctcgagagagcttggtgagcacagagtc
		pT (pPGK)	pPGK-XbaI-F: taatctagataattctaccgggtaggggaggc<br>pPGK-SacII-R: gtggccgcgggaaaatagatctcgaaaggcccggagatgag
		pV2O (V2O gene)	V2O-SacII-F:ttcccgcgggaattcgatgatccgccgccacca tgggcgcgggcg<br>V2O-XhoI-R: ctcctcgagcggttccagttccaggcg
		pT (EGFP gene)	EGFP-NcoI-F: gccaccatggtgagcaagggcgag<br>EGFP-XhoI-R: ctcctcgagatctgagtacttgtacagctcgtccatgccga
N3	mic13lg+mic1002-Puro (AfeI-XhoI)	pV2O (V2O gene)	V2O-NheI-F: actgctagctgtagaactccagcttagatcccgcgactctaggatcc gccgccaccatgggcgcgggc<br>V2O-XhoI-R: ctcctcgagcggttccagttccaggcg
		pT (TRIM5a-HRH)	2a-trim-XhoI-F: ccgctcgaggagggcagaggaagtcttctaacatgcggtgacgtg gaggagaatcccggccctggtaccgcttctggaatcctggttaatg<br>trim-XbaI-R: tatctagatcaagagcttggtgagcacagagtc

		pT (pPGK)	pPGK-XbaI-F: taatctagataattctaccgggtaggggaggc<br>pPGK-NcoI-R:<br>caccatggtggcgaccggtggatctcgaaaggcccggagatgag
		pT (EGFP gene)	2a-EGFP-XhoI-F:<br>tctctcgaggagggcagaggaagtcttctaacatgcggtgacgtgg<br>aggagaatcccggccctggtaccgtgagcaagggcgaggag<br>EGFP-NotI-R:<br>gacgcggccgccttgtacagctcgtccatgccga
N4	N3(AfeI -XbaI)	pT<br>(TRIM5a-HRH)	Trim-AfeI-F: gccagcgctgccaccatggcttctggaatcctg<br>trim-Xho-R: ctcctcgagagagcttggtgagcacagagtc
		pV2O (V2O gene)	2a-V2O-XhoI-F:<br>tctctcgaggagggcagaggaagtcttctaacatgcggtgacgtgg<br>aggagaatcccggccctggtaccggcgcgggcgcgaccggccg<br>c<br>V2O-XbaI-R: ttatctagattacggttccagttccaggcgttc
N5	N1(XbaI-NotI)	pT (pPGK)	pPGK-XbaI-F: taatctagataattctaccgggtaggggaggc<br>pPGK-NcoI-R:<br>caccatggtggcgaccggtggatctcgaaaggcccggagatgag
		pT (EGFP gene)	2a-EGFP-XhoI-F:<br>tctctcgaggagggcagaggaagtcttctaacatgcggtgacgtgg<br>aggagaatcccggccctggtaccgtgagcaagggcgaggag<br>EGFP-NotI-R:<br>gacgcggccgccttgtacagctcgtccatgccga
		pT<br>(TRIM5a-HRH)	2a-trim-XhoI-F:<br>ccgctcgaggagggcagaggaagtcttctaacatgcggtgacgtg<br>gaggagaatcccggccctggtaccgcttctggaatcctggttaatg<br>trim-NotI-R:<br>gacgcggccgctctagatcaagagcttggtgagcacagagtc
N6	N2(XbaI-NotI)	pT (pPGK)	pPGK-XbaI-F: taatctagataattctaccgggtaggggaggc<br>pPGK-NcoI-R:<br>caccatggtggcgaccggtggatctcgaaaggcccggagatgag
		pT (EGFP gene)	2a-EGFP-XhoI-F:<br>tctctcgaggagggcagaggaagtcttctaacatgcggtgacgtgg<br>aggagaatcccggccctggtaccgtgagcaagggcgaggag<br>EGFP-NotI-R:<br>gacgcggccgccttgtacagctcgtccatgccga
		pV2O (V2O gene)	2a-V2O-XhoI-F:<br>tctctcgaggagggcagaggaagtcttctaacatgcggtgacgtgg<br>aggagaatcccggccctggtaccggcgcgggcgcgaccggccg<br>c<br>V2O-NotI-R:

			gacgcggccgctctagattacggttccagttccaggcg

A B

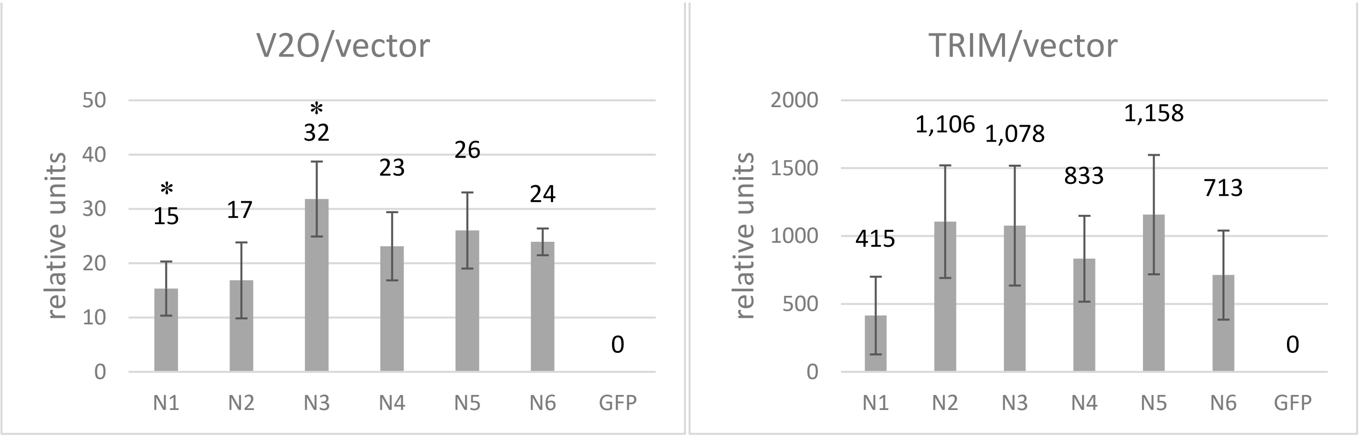

Figure S1 Estimation of the mRNA level of the V2O and TRIM5a-HRH genes. The cells were transduced with various lentivectors, after 3 weeks the cells were collected, treated with benzonase, then mRNA was isolated and OT-qPCR was performed with primers to the V2O(A) and TRIM(B) genes. Normalization was performed on the average number of vector per cell, which was determined using qPCR as WPRE normalized to Globin. * p≤0.05
